# Dynamic Analysis of Gray Matter and White Matter Functional Network Connectivity in Individuals with Temporal Lobe Epilepsy

**DOI:** 10.21203/rs.3.rs-8206698/v1

**Published:** 2026-01-12

**Authors:** Sukesh Das, George B Hanna, Hai Sun, Bharat B. Biswal

**Affiliations:** 1 Department of Biomedical Engineering, New Jersey Institute of Technology, Newark, NJ 07102, New Jersey, USA.; 2 Department of Neurosurgery, Rutgers Robert Wood Johnson Medical School, New Brunswick, NJ 08901, New Jersey, USA.

**Keywords:** Temporal lobe epilepsy (TLE), White matter (WM), Resting state functional magnetic resonance imaging (rs-fMRI), Dynamic functional connectivity (dFC), Functional network connectivity (dFNC), Dynamic functional connectivity states

## Abstract

Accurately localizing the epileptic zone in temporal lobe epilepsy (TLE) is challenging. Emergent resting-state functional connectivity (rs-FC) analyses have demonstrated that the epileptic network of TLE likely involves both gray (GM) and white matter (WM). Clustering dynamic functional network connectivities (dFNCs) reveals time-varying dependencies of functional networks (FNs) and may provide additional insight into TLE, as interictal discharges are often transient and spread across FNs. In this study, we analyzed data from **103** participants from the Epilepsy Connectome Project (ECP), including **51** healthy controls, **34** individuals with left TLE, and **18** with right TLE. We used K-means clustering to derive FNs in GM and WM. We calculated static and dynamic FNC between these FNs, using the average correlation for static FNC and standard deviation (STD) for dynamic FNC. We then obtained dynamic FNC states by clustering windowed FNCs. We did not find any significant connections in dynamic FNC (dFNC) using STD. However, dFNC states demonstrated the association of WM FNs, altering connectivity in TLE. The dynamic state that was slightly negatively correlated with the sFNC showed significant changes in TLE, affecting not only GM but also the GM-WM and WM-WM connections. Using dFNC, we observed notable connections that were absent in both higher negative and positive FNC patterns and did not fall within the strongest **1**% connections. Furthermore, a significant GM-WM connection was found in a state in which the probability of self-transition differs between TLE and HC. The state analysis (dynamic) reveals that the FCs between temporooccipital - lateral occipital cortex and Superior frontal gyrus - left frontal orbital cortex - paracingulate gyrus, Superior parietal lobule - postcentral gyrus - precentral gyrus and corpus callosum - forceps minor - superior and anterior corona radiata - right superior longitudinal fasciculus - left temporal sub gyral, and left inferior longitudinal fasciculus and hippocampus increased significantly in TLE. The FCs between right angular gyrus - right middle frontal gyrus -right middle temporal gyrus and left inferior longitudinal fasciculus, and right angular gyrus - right middle frontal gyrus -right middle temporal gyrus and left temporal sub gyral - left superior longitudinal fasciculus - left splenium of the corpus callosum decreased significantly. These increase and decrease exhibit only in a low negative connectivity state in TLE. In general, our findings suggest that including WM along with GM FNs and considering time-varying network interactions are important to understanding TLE.

## 1 Introduction

Temporal lobe epilepsy (TLE) is the most common form of pharmacoresistant focal epilepsy in adults. Surgical treatment can often lead to seizure freedom (Téllez-Zenteno and Hernández-Ronquillo (2012); Fallahi et al. (2020); Larivière et al. (2020)). However, surgery can not be performed in approximately 30% of the individuals with TLE due to a lack of clear localization of the epileptogenic zone (EZ) (Berg et al. (2003)). TLE is classified into two main forms: 1. Mesial temporal lobe epilepsy (mTLE), which accounts for about 90% of temporal lobe seizures and involves the mesial temporal structures with seizure activity often originating from a hippocampal or parahippocampal focus. 2. Neocortical TLE (nTLE), where the epileptic focus is located in the lateral temporal lobe (Kennedy and Schuele (2012); Tatum (2012); Téllez-Zenteno and Hernández-Ronquillo (2012)). MRI volumetric analysis, such as voxel-based morphometry, demonstrated atrophy in both the temporal and extratemporal grey and white matter (GM and WM) (McMillan et al. (2004); Mueller et al. (2006)). In addition to these structural alterations, functional alterations have been shown to cause widespread changes extending to the limbic structures (e.g., the entorhinal and perirhinal cortices and the amygdala), subcortical regions (e.g., the thalamus), and extratemporal areas (e.g., the frontal lobes and the superior, middle, and inferior temporal gyri) (Lieb et al. (1991); Spencer and Spencer (1994); Spencer (2002); Bartolomei et al. (2004); Laufs et al. (2007); Haneef et al. (2012); Dabbs et al. (2012); Cataldi et al. (2013); Liao et al. (2010); Hermann et al. (1991); Giovagnoli (2001); Mueller et al. (2004)). Although TLE has traditionally been considered a focal disorder, it is increasingly considered a network disease.

Low-frequency fluctuations in resting-state functional magnetic resonance imaging (rs-fMRI) signals, which have physiological significance and are thought to reflect ongoing neuronal activity (Biswal et al. (1995); Lu et al. (2007)), are increasingly being explored as a promising approach to studying intrinsic functional properties of the brain (Guo et al. (2013); Barkhof et al. (2014)). fMRI acquired during the task-free conditions can delineate the brain network dynamics and neural synchronization of resting-state networks (RSNs) across various diseases, including TLE (Seeley et al. (2009); Jiang et al. (2019a,b); Bettus et al. (2009); Haneef et al. (2014); Pittau et al. (2012); Liao et al. (2010); Zhang et al. (2010); Cataldi et al. (2013)). A study by Su and colleagues indicated that patients with mTLE with right hippocampal sclerosis exhibited decreased resting state functional connectivity (rs-FC) within the right hemisphere and increased rs-FC within the left hemisphere (Su et al. (2015). These findings suggest that right mTLE may have weakened the rs-FC in the right hemisphere and strengthened rs-FC on the contralateral side, which may be related to the seizure-induced damage and underlying compensatory mechanisms (Fallahi et al. (2020)). The default mode network (DMN) is composed of brain regions involved in conscious, resting state cognition. The hippocampus is an essential component of this network. Connectivity between posterior and anterior regions of the DMN was reduced in the patients with TLE, regardless of laterality of EZ (Haneef et al. (2012)) but their individual connections remain preserved.

Examining the functional interactions between different brain networks has become a powerful method for studying brain function at rest, offering a potential avenue for diagnosing neuropsychiatric diseases. The temporal dependency among these functional networks (FNs) is called functional network connectivity (FNC) (Jafri et al. (2008)). As in TLE, the propagation of epileptic activation might involve multiple functionally connected regions, extending to GM as well as WM, which differ from the HC. Both GM and WM analyses have received significant attention in TLE (Jiang et al. (2019b); Cui et al. (2021); Li et al. (2021)). As a result, the investigation of FNC (including GM and WM) may reveal intriguing insights into the pathophysiological mechanism of TLE (Liu et al. (2017)). Typically, static FNC (sFNC) is calculated using the whole scanning period with the assumption that FC remains stationary during the observation period; however, it actually represents a measure of average different dynamic modes of brain activity during an unconstrained resting state (Damaraju et al. (2014); Li et al. (2015, 2016); Liu et al. (2017)). Hence, it may be less sensitive when discriminating between groups (Liang et al. (2021); Wang et al. (2016)) and might not be enough to fully characterize the dynamically changing associations between networks (Van Dijk et al. (2010); Deco et al. (2010); Hutchison et al. (2013)). The time-dependent nature of the brain activity drives FC changes over time (Allen et al. (2012); Damaraju et al. (2014)) and this dynamic functional connectivity (dFC) can better encode the variations in clinical cohorts (Calhoun et al. (2014); Kopell et al. (2014); Kucyi and Davis (2015)). Typically, the dynamic method is computed using a sliding temporal window to estimate FNC over shorter time intervals, and it can designate multiple discrete network configurations, also referred to as states, occurring throughout the entire resting-state scanning period and can characterize dynamic changes across different groups (Liang et al. (2021)).

Liu and colleagues revealed state-specific FNC disruptions in Idiopathic generalized epilepsy (IGE) characterized by generalized tonic-clonic seizures (GTCS) (Liu et al. (2017)). A similar analysis was also carried out to uncover the network alteration associated with TLE progression (Liang et al. (2021)). This study showed that short-duration TLE spent more time in a strongly connected dynamic FNC (dFNC) state and showed greater inconsistent abnormal network connectivity than long-duration TLE. In another study, state-specific dFNC analysis demonstrated a decreased connection between the subcortical nuclei and frontoparietal network in frontal lobe epilepsy (Klugah-Brown et al. (2018)). Though these studies have performed dynamic state analysis on dFNCs, they derived the FNs from the whole brain data (GM and WM) using independent component analysis (ICA) (Calhoun et al. (2001)). While ICA has been shown to be a powerful data-driven method based on BOLD time courses for obtaining FNs, it requires the initialization of independent spatial sources (Venkataraman et al. (2009)). Recently, an FC-based multivariate clustering method has been used to derive reliable FNs (Peer et al. (2017)).

In this study, the dynamic dependencies among GM–GM, WM–WM, and GM–WM were investigated using standard deviation and dynamic state centers. FNs were derived separately using GM and WM signals to examine whether their interactions (i.e., GM–GM, WM–WM, and GM–WM) had any impact on specific dFNC states during the scan. The GM and WM FNs were obtained using multivariate clustering, which involves applying a K-means algorithm to the parcellated FC (Schaefer 400 (Schaefer et al. (2017)), and the Eve atlas (Type III WM parcellation map) (Oishi et al. (2009)), respectively). K-means clustering was performed on the ROI-based static FC (sFC), and the FNs were obtained. The average time courses were extracted from each GM and WM network, and dFNC was computed for every sliding window and repeated over time. Since interictal epileptic discharges are often transient and spread across different brain regions, affecting multiple networks in epilepsy, dFNC analysis may provide additional insight into the time varying dependency of FNs in TLE (Klugah-Brown et al. (2018); Abreu et al. (2019)). In this study, dFNCs across the sliding windows from all subjects were then grouped into discrete states using K-means clustering, and state analysis (dynamic) was performed between TLE and HC groups. The dFNC states exhibit more interactive FC among the FNs at higher temporal resolution and can be used to investigate network alterations among patients with TLE. Primary goal of this study was to assess the difference between the static connectivity and the state-specific dynamic connectivity among GM and WM-FNs. The state-specific analysis was performed to pinpoint the alteration between HC and TLE at the state level, i.e., during some specific duration, and different temporal characteristics of states were investigated. Overall, this study will shed light on the alteration of the FNs (both GM and WM) during the resting state in both the temporal and spatial domains.

## 2 Method

### 2.1 Participants

We used publicly available data from the epilepsy connectome project (ECP) ^1^. The ECP study had a total of 236 subjects, and for this study, only data sets (196 subjects) from the first session (Run 1) that had both T1 and resting state fMRI data available were used. Out of 196 subjects, we considered 103 subjects (64 females and 39 males, average age 36.73 years) with 52 unilateral TLE and 51 healthy controls (HC). Out of the 52 TLE subjects, 34 and 18 subjects had their seizure on the left and right side, respectively. The mean and standard deviation of the ages of the HC and TLE groups are 34.41 ± 11.10 and 37.28 ± 12.16.

### 2.2 Image acquisition

All the data from ECP subjects were acquired on standard GE Healthcare Discovery MR750 MRI systems (3T) housed at the Medical College of Wisconsin and the University of Wisconsin at Madison. The integrated body RF coil was used for exciting, while a Nova Medical 32-channel receive only head coil was used for signal reception. T1-weighted structural images were acquired using a three-dimensional gradient-echo pulse (MPRAGE) sequence with the following parameters: repetition time (TR)=604 ms, echo time (TE)=2.516 ms, flip angle=8 degree, field of view (FOV)=25.6 cm, voxel size = 0.8 mm (isotropic). Resting-state functional MRI (rs-fMRI) data were acquired using a gradient recalled echo (GRE), echo planar imaging (EPI) sequence with the following parameters: TR=802 ms, TE=33.5 ms, FOV=20.8 cm, flip angle=50 degree, matrix=104*×*104, number of slices=72, voxel size=2 mm (isotropic), multiband acceleration factor of 8. Two sessions, each containing a set of four 5-min resting state scans (axial acquisitions), were acquired in pairs of runs which alternated anterior-to-posterior (AP) versus posterior-to-anterior (PA) phase encoding in directions, totaling eight scans. Participants were asked to gaze at a white cross on a black background. For every subject, we considered 361 time points in our study.

### 2.3 Data Pre-processing

All the data across each of the subjects was pre-processed as follows: 1) The first 10 volumes were discarded to ensure steady-state longitudinal magnetization, 2) The functional images were realigned, 3) Subjects with maximum displacements greater than 2 mm or 2 degrees were excluded from further analysis, 4) Individual T1 images were segmented into GM, WM, and CSF to obtain the tissue probability map transformation from native to standard MNI space. The resulting segmented images were coregistered to the functional space for each participant, 5) The nuisance signal (including 24 motion parameters (6 rigid body head motion parameters at the current time point, 6 parameters at previous time points, and the 12 corresponding squared values) and the mean cerebrospinal fluid (CSF) time course were regressed out from all the voxel time series. 6) Temporal filtering (using 5th-order Butterworth) was done in the low-frequency range of 0.01 – 0.15 Hz to reduce non-neuronal contribution to BOLD fluctuations (Peer et al. (2017)). 7) To minimize the mixing of the GM and WM signals, individual functional images were spatially smoothed with an isotropic kernel of full width at half maximum (FWHM) of 4*mm* × 4*mm* × 4*mm* separately within the GM and WM masks (Peer et al. (2017)). Then, GM and WM images were merged into full functional images. 8) Finally, the functional images were normalized to the standard MNI template with a voxel size of 2*mm* × 2*mm* × 2*mm*. The entire preprocessing task was performed using SPM12 ^2^ and in-house MATLAB (R2023a) scripts.

### 2.4 Dataset and code availability

ECP data is publicly available at https://osf.io/exbt4/. The codes for deriving the FNs and dFNC analysis are available at https://github.com/sentudas32/dFNCStates.

### 2.5 Deriving GM and WM networks using FC

**Fig. 1:**
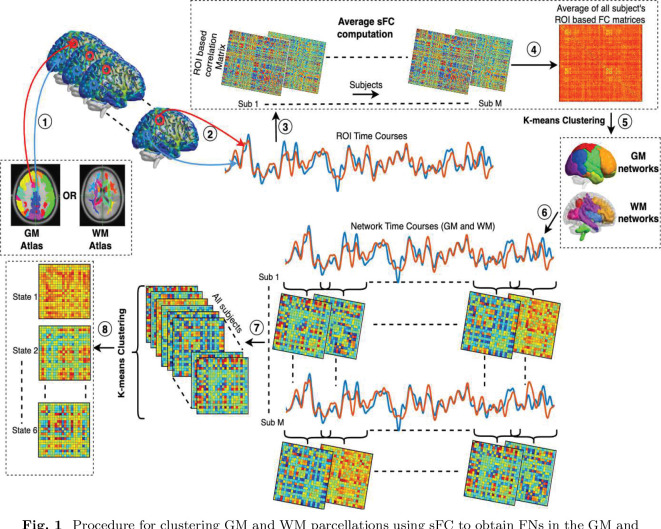
Procedure for clustering GM and WM parcellations using sFC to obtain FNs in the GM and WM, and dFNC state analysis. The processing steps are as follows: (1) Time courses are extracted from ROIs of the GM (Schaefer 400) and WM atlas (Eve atlas). (2) Average ROI time courses were computed from GM and WM regions, respectively. Figs 1.1 - 1.4 demonstrate the computation of average sFC in GM and WM, respectively. (3) Subject-wise ROI-based correlation (sFC) matrices were computed. (4) sFC matrices are averaged across all subjects (M), and an average FC was obtained. (5) A K-means clustering was performed on the averaged sFC matrix using correlation as the distance metric and with 50 replications. The optimal value of K was found using 8-fold cross-validation with adjacency matrices as described in Section 2.5. K number of clusters (FNs) were derived from the K-means clustering using the optimal value of K with the lowest distortion from 100 replications. (6) Average time courses were extracted from all clusters (GM-FNs and WM-FNs). Dynamic FCs (dFCs) between the FNs (GM and WM) were computed using Pearson’s correlation for every subject (*m* =1 to *M*) at every sliding window. Windowed functional connectivities were Fisher’s Z-transformed with sex and age effects being regressed out. (8) All windowed FNCs of all subjects are clustered using the K-means algorithm, and every cluster centroid was designated as a dynamic state of FNC. The optimal number of clusters or states was obtained using the elbow method.

The subject’s static FC of GM and WM regions of interest (ROIs) was clustered respectively using the K-means clustering method to obtain corresponding GM and WM networks. A schematic diagram for clustering the ROI-based sFC has been shown in Figs. 1. A GM parcellation mask (Schaefer 400 (Schaefer et al. (2017))), consisting of 400 cortical ROIs, was used to derive GM FNs, and a WM parcellation mask (Eve atlas, Type III WM parcellation map) (Oishi et al. (2009)), consisting of 128 ROIs, was used. The WM mask is a parcellation of 44 superficially located WM (SWM) and 56 deep WM (DWM) structures. The outline of the SWM is based on the 90% WM probability. This parcellation includes the subcortical GM regions, including the thalamus, putamen (Clarke et al. (2022)), hippocampus, and brainstem, and these regions were also found to be vulnerable to functional alterations in TLE (Hryniewicz et al. (2024); Lucas et al. (2023); Norden and Blumenfeld (2002)); therefore, we included them in our clustering analyses. Average ROI time courses were extracted from the GM and WM regions, subject-wise Pearson’s correlation matrices (sFC) were calculated, and average correlation matrices were obtained across all subjects. Distinct GM and WM functional networks are identified by performing the k-means clustering on the average GM and WM sFC matrices, respectively. In K-means clustering, the distance metric was used as the correlation, and 50 replications were considered. The number of clusters was chosen between 5 and 20. The cluster stability or the optimal number of clusters was achieved based on the number of folds (nf = 8) cross-validation using adjacency matrices (Peer et al. (2017)). The average connectivity matrix was randomly divided into a given nf, and clustering computation was performed on each fold separately. The cluster indices were divided into chunks (chunk size, cs=30) in each of the nf folds. For every pair of chunks, a binary 3D matrix (*cs* × *cs* × *nf*) was formed. Then, an average of the dice coefficients was computed by comparing all nf adjacency matrices for every K with 50 replications. The k that yielded the highest dice coefficients was the optimal k resulting in the stable clusters. Finally, k means clustering was performed using the optimal number of k, and GM and WM clusters (FNs) were obtained with the lowest distortion from 100 replications.

### 2.6 Optimal window size for dFNC

The length of window used for dFNC computation is important, and the dFNC statistics vary based on the window length. If the length of the sliding window is too short, it may raise spurious values; however, if it is too long, it may miss crucial dynamics. Once the FNs (GM and WM) were obtained, the Fisher discriminant ratio (FDR) was computed to perform a discriminant assessment between two individual groups for comparing HC to the TLE group (FISHER (1936); Das et al. (2024, 2025)) at various length of windows. The FDR values were used to determine which window length is most suitable for discriminating between the two groups. It is calculated as the ratio of between-group variance to within-group variance of dFNC (GM and WM), with dFNC computed using standard deviation (STD) as static. Therefore, the mean and variance of the dFNC were computed for HC and TLE, respectively. A high FDR indicates that the two groups are widely separated using the particular length of window. The FDR between HC and TLE for average dFC with a particular window is given by the following equation

(1) $$J=\frac{\left|\mu_{HC}\;-\;\mu_{TLE}\right|}{\sigma_{HC}^{2}\;-\;\sigma_{TLE}^{2}},$$

where, $\mu_{HC}$ and $\sigma_{HC}^{2}$ are the mean and variance of the average (irrespective of FNs) dFNC score (STD) of HC, respectively. $\mu_{TLE}$ and $\sigma_{TLE}^{2}$ are the mean and variance of the average (irrespective of FNs) dFNC score (STD) of TLE, respectively.

### 2.7 Clustering Dynamic functional network connectivities (dFNCs)

An average of the time series across all voxels within each of the GM-FNs and WM-FNs was computed. The FNs were defined by the clusters from the K-means clustering, resulting in *K*1 and *K*2 time courses for each individual cluster in the GM and WM regions respectively. Then, for each subject (HC or TLE), (*K*1 + *K*2) time courses from the FNs were extracted, and the FNC value was obtained using Pearson’s correlation among the FNs over every sliding window. The window length was determined based on the discriminating analysis between the HC and TLE using FDR, as discussed in Section 2.6. The windowed FNCs were Fisher’s Z-transformed, and sex and age effects were regressed out for all subjects. This dFNC signified the FC between the GM - GM FNs, WM - WM FNs, and GM - WM FNs over a shorter period of time. These dFNC patterns recur across time and subjects. Next, the K-means clustering was employed to group the homogenous patterns of the windowed FNC, which repeats within subjects across time ignoring their temporal order. We selected *K* for the number of state selections (*ks*), ranging from 2 to 15, representing cluster states of windowed FNC. For every value of ks, 50 replications were considered with the distance metric as the cosine. The optimal number of states was obtained from the elbow method, which is defined as the ratio of within clusters to between clusters distances (Damaraju et al. (2014)). We determined the optimal number of clusters to be ks, where the distortion was significantly low, and centroids of clusters were called dFNC-states. Centroids with the ks number of clusters give a ks number of dFNC states. This optimal number of dynamic states eventually provides the number of distinct dFNC patterns. The extraction of the network time courses and dFNC analysis have been illustrated in Figs 1.6 - 1.8. Finally, state analysis was conducted to compare the groups.

### 2.8 Individual dynamic FNC states using dual regression

To reconstruct the subject-specific states from the group-level states, we used the dual regression approach as an exploratory method (Beckmann et al. (2009); Klugah-Brown et al. (2018)). In the first regression step, individual FNC matrices (Y) were approximated using the group-level states (S) as a set of spatial predictors, and temporal dynamics ($\beta_{1}$) of FNC associated with each group-level state were estimated (Eq. 2). $\varepsilon_{1}$ denotes the error term. In the second regression step, the estimated dynamics ($\beta_{1}$) of the FNC, obtained in Eq. 2, were set as predictors to estimate subject-specific states ($\beta_{2}$) as shown in Eq. 3, where $\varepsilon_{2}$ denotes the error term. These are seen in the following equations, $\in R^{w\times m}$, $S\in R^{k\times m}$, $\beta_{1}\in R^{k\times w}$, $\beta_{2}\in R^{k\times m}$, and $m=\frac{n(n-1)}{2}$, where n=(K1+K2) is the number of functional networks, k is the number of states, and w is the number of sliding windows for FC computation.

(2) $$Y^{T}=S^{T}\beta_{1}+\varepsilon_{1},$$

(3) $$Y=\beta_{1}^{T}\beta_{2}+\varepsilon_{2},$$

### 2.9 Time frame’s contribution to states

The probabilities of the FNC frames in every state were computed. The probability of the FNC frame for a group in a state was computed as

$$Probability\;of\;FNC\;frame\;for\;a\;group\;A\;in\;a\;state\;=\frac{\;\mathrm{No}\;\mathrm{of}\;\mathrm{FNC}\;\mathrm{frames}\;\mathrm{of}\;A\;\mathrm{in}\;\mathrm{that}\;\mathrm{state}\;}{\;\mathrm{Total}\;\mathrm{no}\;\mathrm{of}\;FNC\;\mathrm{frames}\;\mathrm{of}\;A\;\mathrm{across}\;\mathrm{all}\;\mathrm{states}\;}$$

### 2.10 State transition analysis

State transition analysis was performed using the state transition probability and the state changing probability. The average state transition probability was calculated as

(4) $$S(i,\;j)=\frac{1}{N}\sum_{l=1}^{N}\frac{P(i,\;j)}{P_{i}}=\frac{1}{N}\sum_{l=1}^{N}\frac{n_{l}(i,\;j)}{n_{l}(i)},$$

Where S(i,j) is the average state transition probability from state i to state j,N is the total number of subjects in a group, nl(i,j) is the number of transitions that occur in subject l from state i to state j, and $w_{l}$ is the total number of shifting-windows for subject l. $n_{l}(i)$ is the count of how many times state i occur in subject l. For each subject, the sum of all elements in P(i,j) across all i, and j equals 1. This joint probability is then normalized by the prior probability to obtain the conditional probability for each subject. The state-changing probability was computed as the average ratio of the number of transitions from one state to another to the total number of transitions, including transitions that return to the same state.

## 3 Results

### 3.1 Image quality assurance

Individuals were excluded if their maximum framewise translational/rotational displacement exceeded the voxel size (2*mm*) or if their mean framewise displacement was greater than 0.3*mm*. This led to the exclusion of 40 subjects and resulted in 196 individuals (51 HC and 145 TLE) being included from the first session for further analysis. After excluding individuals with bilateral or those with unknown EZ, we included 52 individuals with TLE, 34 with left TLE and 18 with right TLE, along with 51 HC participants.

### 3.2 Functional networks using FC

**Fig. 2:**
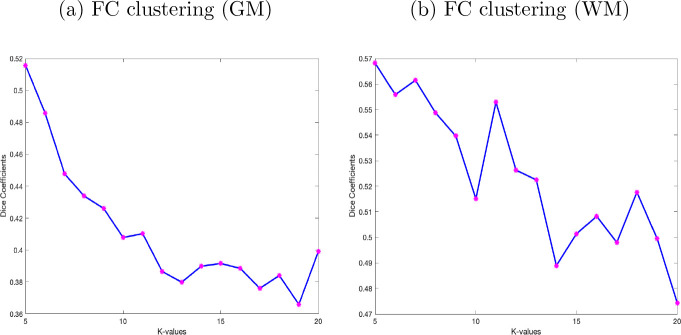
Stability of the clustering FC for different numbers of clusters. The average dice coefficient of the clustering solutions (adjacency matrices) for each number of clusters ranging from *K* = 5 to 20. (a) Dice coefficients from clustering FC in GM. (b) Dice coefficients from clustering FC in WM. For both methods, the optimal value of *K* = 11 was found using 8-fold cross-validation with adjacency matrices.

**Fig. 3:**
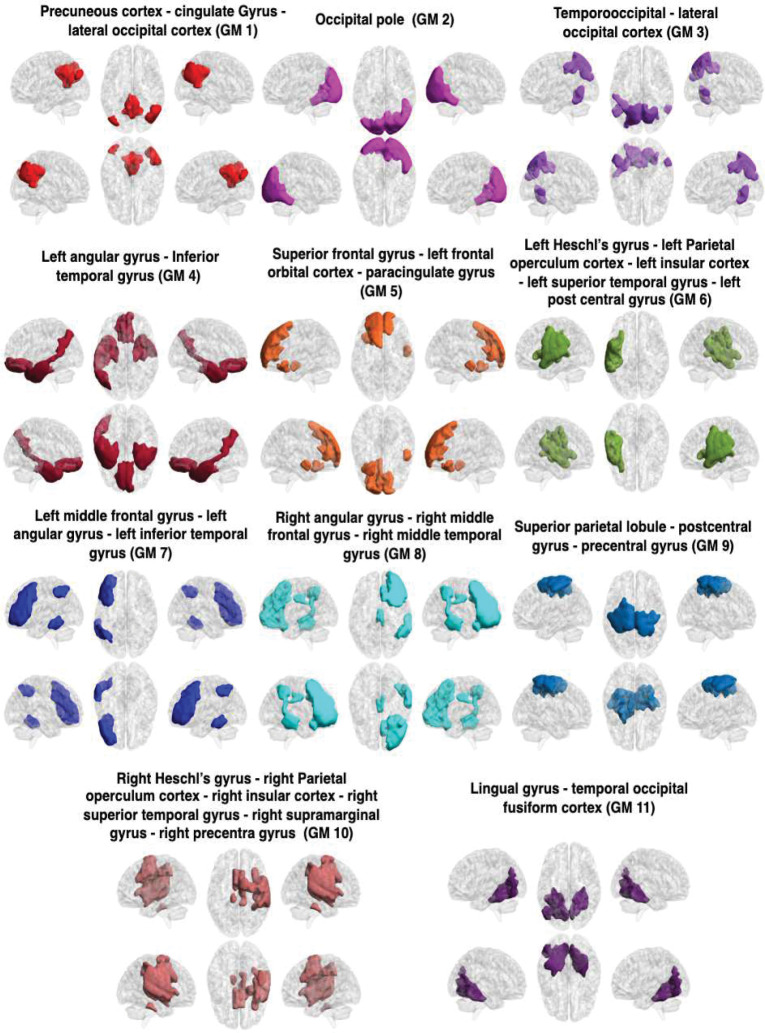
Clusters (FNs) obtained from K-means clustering of the sFC in GM. The GM networks (FNs) are, from left to right and top to bottom: PC - CG - LOC (GM1), OP (GM2), TO - LOC (GM3), lAG - ITG (GM4), ISFG - IFOC - PG (GM5), lHG - lPOC - lIC - lSTG - lPCG (GM6), lMFG - lAG - lITG (GM7), rAG - rMFG - rMTG (GM8), SPL - PoCG - PrCG (GM9), rHG - rPOC - rIC - rSTG - rSMG - rPrCG (GM10), LG - TOFC (GM11). All details of the abbreviations for the regions are provided in Table 1. GM1 represents the default mode network; GM2 represents the visual network. GM3 shows similarity in temporo-occipital connectivity, while GM4 displays frontotemporal connectivity. GM5 is mostly located in the frontal regions. GM6 represents the left auditory connections, and GM7 demonstrates left frontoparietal connectivity. GM8 exhibits right mid-frontal and mid-temporal connectivity. GM9 is associated with sensory regions. GM10 demonstrates right auditory connectivity, and GM11 represents the dorsal attention network.

**Fig. 4:**
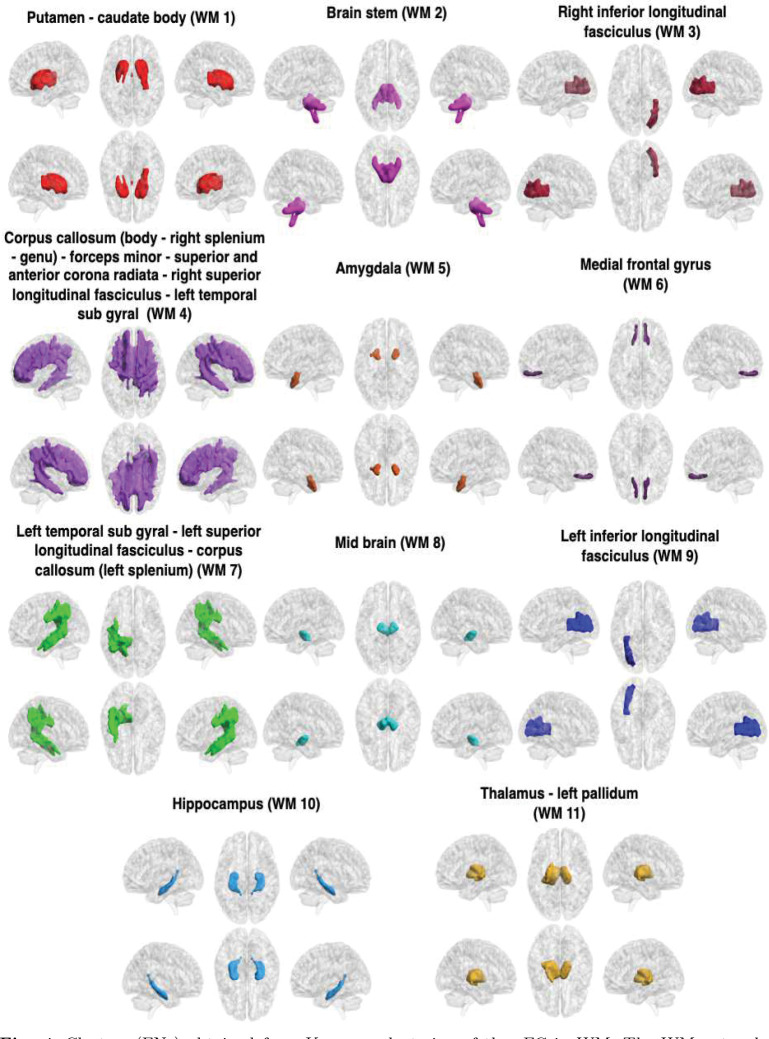
Clusters (FNs) obtained from K-means clustering of the sFC in WM. The WM networks (FNs) are, from left to right and top to bottom: P - CB (WM1), BS (WM2), rILF (WM3), CC(B) - rCC(S) - CC(G) - FM - SACR - rSLF - lTSG (WM4), A (WM5), MFG (WM6), lTSG - lSLF - CC(lS) (WM7), MB (WM8), lILF (WM9), H (WM10), and T - lPa (WM11). All details of the abbreviations for the regions are provided in Table 1. WM1, WM4, WM5, WM6, WM10, and WM11 are mostly located in the superficial layers. Most of WM2, WM7, and WM4, as well as some parts of WM3 and WM9, are found in the middle layer. WM8, along with most of WM3 and WM9, and a portion of WM2, are located in the deep layer.

The GM-FNs and WM-FNs were obtained by performing K-means clustering approaches on an atlas-based sFC. The optimal number of clusters, i.e., *K*, was achieved by maximizing the Dice coefficient between the adjacency matrices using the 8-fold cross-validation. Although clustering similarity naturally decreases with a larger number of clusters, the identified local peak indicates the optimal cluster, leading to low cluster distortion Peer et al. (2017); Jain et al. (2025). The dice coefficients for different numbers of clusters in GM, are shown in Figs. 2(a). It can be observed that the dice coefficient attained its optimal value of 0.412 at *K* = 11 in GM. The optimal value of K was used to derive the final clusters (FNs) in GM. The resulting K clusters are presented in Fig. 3. The WM-FNs were obtained by performing K-means clustering on average ROI-wise FC within the WM parcellations. The optimal number of clusters was also obtained by maximizing the dice coefficient between the adjacency matrices in 8-fold cross-validation. Fig. 2(b) demonstrates the dice coefficients at different values of *K*. WM exhibits higher cluster stability at *K* = 11 with a dice coefficient of 0.556. The Dice coefficient is higher in WM than in GM, and this may be because the number of ROIs is greater in GM than in WM. The resulting clusters, obtained from the K-means clustering of the FC in WM, have been presented in Fig. 4. The FNs in the GM and WM were respectively labelled as GM1, GM2 to GM11 and WM1, WM2 to WM11. The FNs (GM and WM) and regions (with abbreviations) belonging to each network are also shown in Table 1.

### 3.3 Optimal window size and optimal number of states for dFNC

**Fig. 5:**
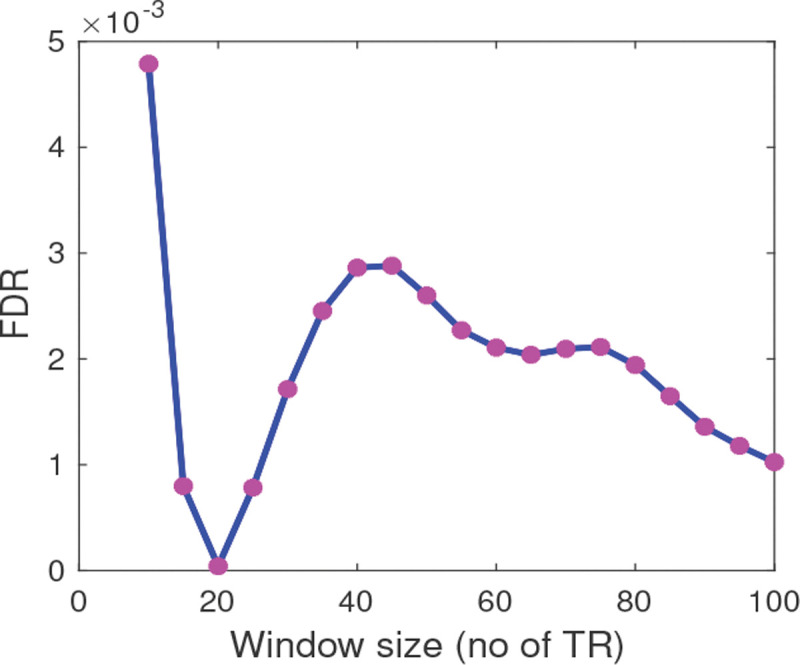
Optimal size of the sliding window for dFNC state analysis. FDR was computed using the standard deviation (STD) of the windowed FNC for every window length starting from 10 TR to 100 TR with a step size of 5 TR. The maximum FDR was observed at a window size of 45 TR.

When searching for the optimal length of the window, we conducted a grid search to maximize the FDR value (using STD as a dynamic statistic), differentiating the HC and TLE groups as described in Subsection 2.6. The FDR values at different windows are shown in Fig. 5. It can be observed that the FDR value decreases from 5 TR to 20 TR and then starts increasing. At a window length of 45 TR, the FDR reaches a local peak (0.028), effectively discriminating between the HC and TLE groups. This window length is also consistent with previous work Allen et al. (2012). We used this optimal window size for computing dFNCs. For computing the optimal number of states, ratios of within-cluster to between-cluster distance at different values of ks (ranging from 2 to 15) were illustrated in supplementary material (Fig. S1). The elbow criterion determined the optimal number of clusters at *ks* = 6.

### 3.4 sFNC and dFNC with standard deviation as a statistic

**Fig. 6:**
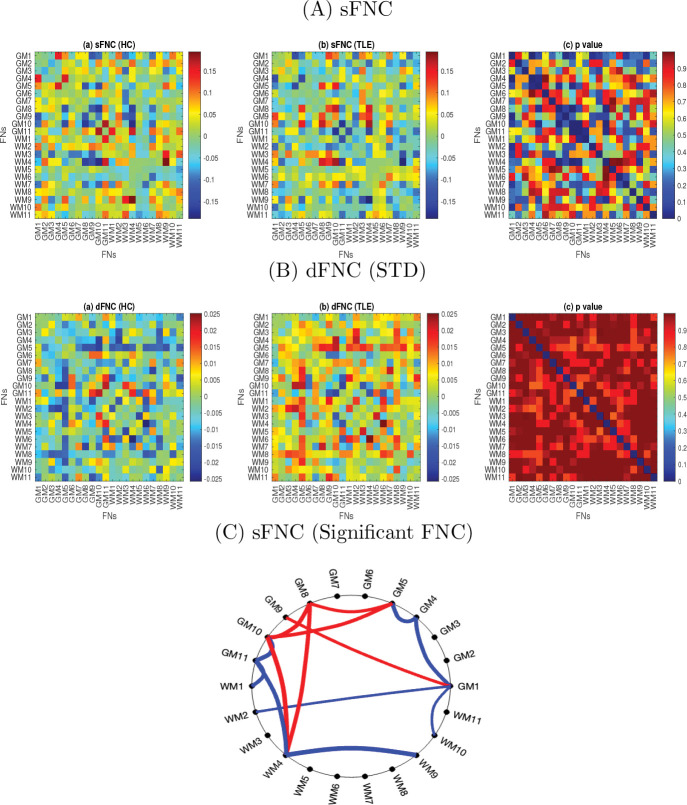
sFNC and dFNC as standard deviation a statistic. A. Average sFNC matrix of the (a) HC, (b) TLE, and (c) the corresponding p values after two-sample t-tests with FDR correction using the Benjamini-Hochberg method. B. Average dFNC matrix of the (a) HC, (b) TLE, and (c) corresponding p values after two-sample t-tests. C. Significant dynamic connection (STD) after two-sample t-test. There are a total of 11 GM FNs and 11 WM FNs. The lines connecting the small solid circles represent the significantly altered connection between FNs. The blue lines represent the significantly reduced fluctuation in the connection between WM2 and WM9

The sFNC among the FNs were computed using Pearson’s correlation for HC and TLE groups, and the averaged sFNC for each group is shown in Fig. 6A(a - b), respectively. All the FCs were Fisher z-transformed, with age and gender effects regressed out. The averaged sFNC was found to be reduced in patients with TLE. The p-values of the FNCs, obtained after two-sample t-tests, were FDR-corrected using the Benjamini-Hochberg method (Benjamini and Hochberg (1995)) and shown in Fig. 6A (c). We observed 8 significantly reduced connections (3 GM-GM, 4 GM-WM and 1 WM-WM connections) and 6 significantly increased connections (4 GM-GM, and 2 GM-WM connections)between FNs. The significantly (p¡0.05) altered connections are illustrated using a circular graph in Fig. 6C. The dFNC with STD of the FNCs across windows were computed, and the averaged dFNCs across subjects are shown in Figs. 6B(a-b) for the HC and TLE groups, respectively. The corresponding p-values were demonstrated in Fig. 6B(c). We did not observe any significant connections between FNs.

### 3.5 dFNC states:

**Fig. 7:**
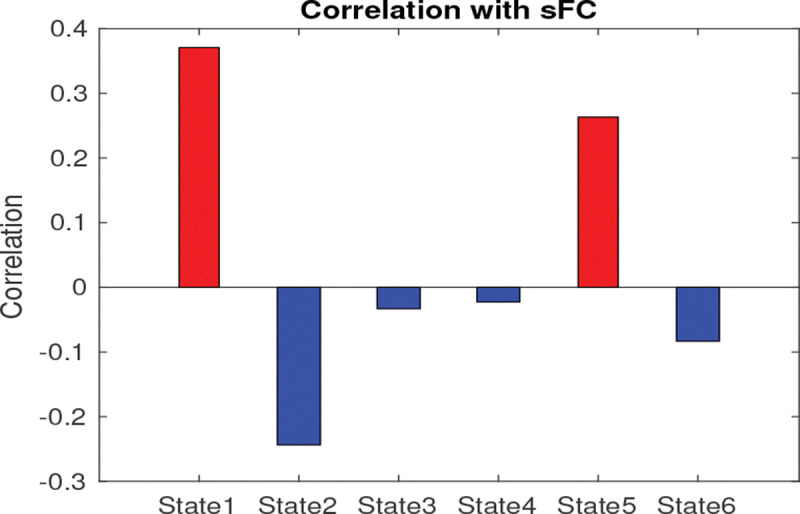
Correlations between sFNC and dFNC states. The red bar represents a positive correlation and the blue bar denotes a negative correlation.

**Fig. 8:**
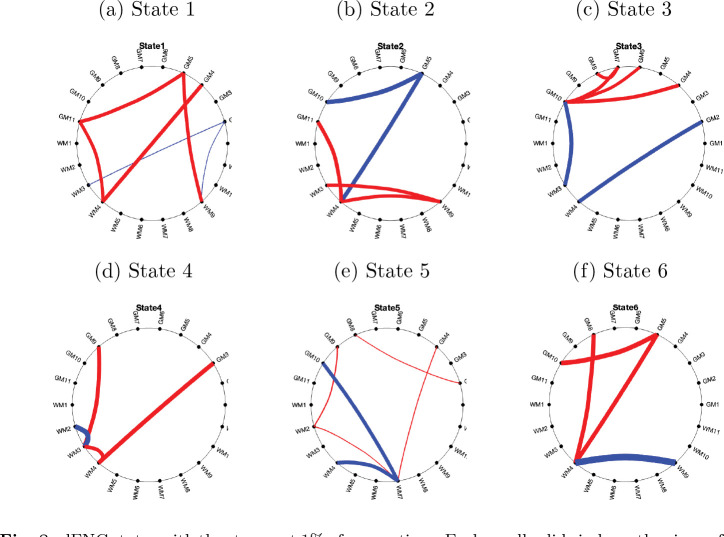
dFNC states with the strongest 1% of connections. Each small solid circle on the circumference of the big circle represents each FN (GM and WM). There are a total of 11 GM-FNs and 11 WM-FNs. The lines connecting the small solid circles represent the connections between the corresponding two FNs. Red lines represent positive connections, and blue lines represent negative connections in each state.

**Fig. 9:**
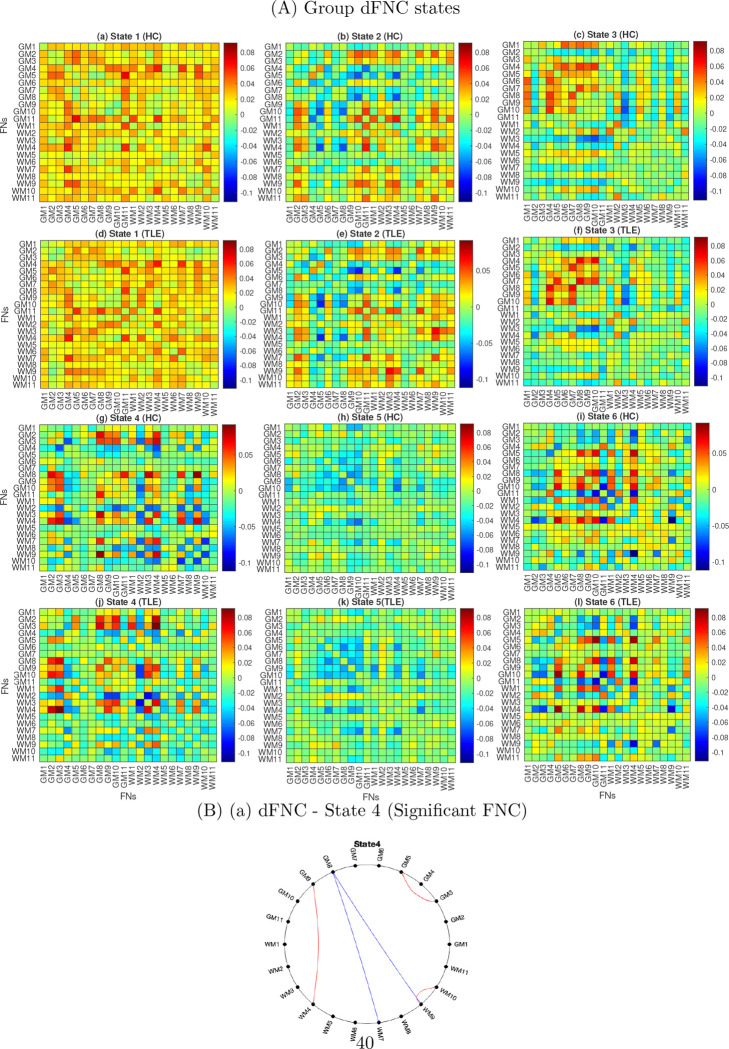
Group-wise dFNC states and significant connections. A. dFNCs are obtained by computing FCs among networks (GM and WM) in each sliding window. State patterns of each group are obtained using K-means clustering of all the windowed FNC across time and subjects. Subject-specific states are computed using dual regression, and group-specific states are computed by averaging the subject-specific states for every group (HC and TLE). B. Significant connections are obtained after two-sample t-tests (state 4). The red color of the connection between the FNs denotes increased connection in TLE, and the blue color of the connection between the FNs denotes decreased connection in TLE.

Irrespective of the groups, the patterns of FNC across the six states are illustrated in supplementary Fig. S2. It can be observed that state 1 was dominated by higher average positive FNC patterns, while state 5 exhibited relatively higher average negative connectivity patterns. The states 2, 6, 3 and 4 displayed relatively low average negative FNC patterns. The state 1 represents the dominant GM - WM connectivity (positive and negative), and the state 3 represents the strong GM - GM connectivity. The state 6 represents the average positive dominating GM - WM connectivity and the dominant negative WM - WM connectivity. The correlation between dFNC states and sFNC has been illustrated in Fig. 7. States 1, and 5 show a positive correlation, and states 2, 3, 4 and 6 show a negative correlation with the sFNC. The state 1 shows a high positive correlation and the state 4 shows a high negative correlation with sFNC. The strongest 1% of FNC connections have been demonstrated in the circular graph (Fig. 8). The strongest positive connections were observed in states 1, 3 and 6. The strongest negative connections were observed in states 6, and 2. Overall, the strongest positive connections outnumbered the strongest negative connections, with the strongest positive connections predominantly observed in GM as well as in WM-FNs. The strongest positive connection was observed between GM5 and GM10 (0.60) in state 6 and between GM3 and WM4 (0.5598) in state 4. The strongest negative connections between WM4 and WM9 were observed in the states 6. We obtained subject-specific states using dual regression as described in Section 2.8, and group-specific states were computed by averaging the subject-specific states of two different groups (HC and TLE). The six distinct FNCs states obtained across two groups are demonstrated in Fig. 9A, and the corresponding significant FNCs are shown in the circular graph (Fig. 9 B). Figs. 9A (a - c, g - i) demonstrate the states corresponding to the HC, and Figs. 9A (d - f, j - l) demonstrate the states corresponding to the TLE group. The average FNC decreased in state 3 and increased in states 2, 5, and 6 in the TLE group. Five significantly altered connections were observed in state 4 after a two-sample t-test and FDR correction using the Benjamini-Hochberg method (Fig. 9 B). The connections, GM3 - GM5, GM9 - WM4, and WM9 - WM10 significantly increased in the individuals with TLE in state 4, while the connections, GM8 - WM7 and GM8 - WM9 significantly decreased in the individuals with TLE in same state (4).

#### 3.5.1 Subject’s and time frame’s contribution to states

**Fig. 10:**
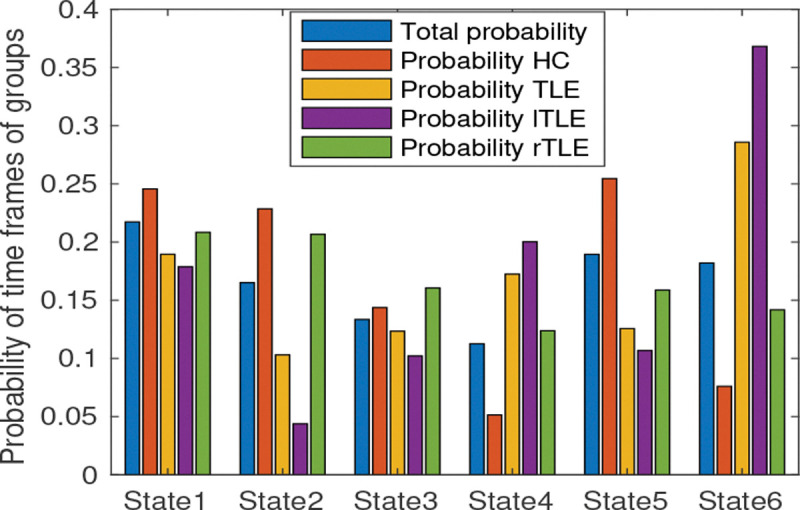
Probability of FNC frames of different groups. Blue, orange, yellow, and green bars represent the probability of all subjects, healthy control subjects, left TLE subjects, and right TLE subjects, respectively.

The probability of time frames of groups is illustrated in Fig. 10. The probabilities were computed using the equation described in Section 2.9. It can be seen that the total probability is high in states 1, 5, and 6 for all groups. The probability of the FNC frames of HC is highest in state 5 and lowest in state 4 whereas the probability of the TLE is highest in state 6 and lowest in state 2.

#### 3.5.2 State transition probability

**Fig. 11:**
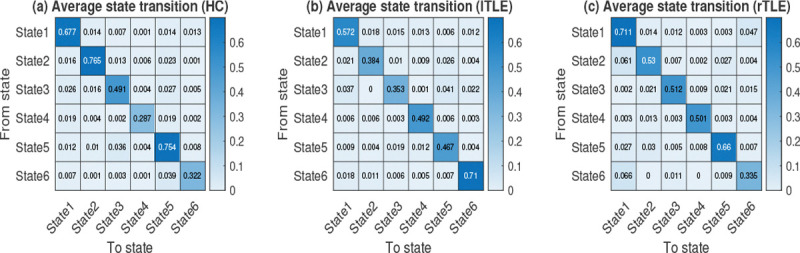
Average state transition probability matrices of (a) HC, (b) left TLE, and (c) right TLE. The transition probabilities (or conditional probabilities) were calculated by dividing the joint probabilities by the prior probabilities of the different states.

The state transition probabilities were computed as described in Section 2.10 and demonstrated in Fig. 11. It can be observed that a dFNC frame, when in a state, does not immediately transition out but stays in that state for a longer duration. In state 2, the HC group exhibits longer dwell times, whereas the lTLE group remains longer in state 6, showing the greatest divergence from the rTLE group in this state. The most prominent differences in state transition probabilities are observed in states 6 and 2. State 4 shows moderate differences between HC and both lTLE and rTLE groups.

State transition probabilities are provided in the supplementary material (Fig.S3). As shown in Fig. S3, the HC group has a higher state-changing probability compared to the TLE group, in both lTLE and rTLE groups. The average state-changing probabilities for HC, lTLE, and rTLE are 0.037, 0.032, and 0.033, respectively.

## 4 Discussion

In this study, we analyze dFNC to investigate the alteration of resting state GM and WM networks in individuals with TLE. It is a crucial approach for statistically identifying alterations in network connections in neuropsychiatric disorders (Jafri et al. (2008); Wang et al. (2015)). The dFNCs were analyzed using the properties of dFNC states including the centroid of clusters, state transition probability, degree of similarity of the states with sFNC, and group contribution to the states. Significantly altered FNC were found during dFNC analysis, i.e., in states that were slightly negatively correlated with sFNC (Fig. 7 and Fig. 9B). The reason may be that the sFNC is similar to the FNC in states 1, and 5, which are not very discriminative. Average (static) FNC is more sensitive than the dFNC (STD) at capturing group differences (Fig. 6A). In the current study, the dFNC(STD) analysis did not show any significant group differences (Fig. 6A) and it explored FNC matrices obtained from different temporal intervals to uncover state-specific alterations in TLE. The study resulted in the following findings: 1) compared with the healthy subjects, TLE patients exhibited reduced connectivity in state 3 but increased connectivity in states 2, 5, and 6. Significantly increased connectivity was observed in connections, GM3 - GM5, GM9 - WM4 and WM9 - WM10 in state 4, and significantly decreased connectivity was observed in connections, GM8 - WM7 and GM8 - WM9 in state 4, respectively. 2) HC exhibits lowest intra-state transition in state 4 but TLE shows moderate transition. The method, therefore, explores a novel insight into the fundamental pathophysiological mechanisms in TLE.

The sFNC analysis, based on the hypothesis that network configurations are stationary in nature and reveal only significantly altered average connectivity patterns in TLE. However, it is crucial to determine whether this alteration or no alteration persists over time during the resting state or arises only during specific brain network configurations. Furthermore, the consistency or inconsistency between groups in FNCs could well be attributed to the mental states during the scanning and, therefore, could well be investigated using this state analysis (Damaraju et al. (2014)). Current study identified different network configurations or state patterns using dynamic analysis based on k-means clustering on the sliding windowed FNCs to investigate the alteration of FNC in TLE from HC. The states are FNC configurations that recur over time. Six dFNC state-patterns were identified across time and subjects. The evaluations were carried out within temporal and spatial domains of the subject’s dFNC patterns. The most pronounced abnormalities were observed during states 1 and 5.

The average sFNC decreased in TLE (Fig. 6A), while the average dFNC (STD), representing fluctuations in the connections between FNs, increased in TLE (Fig. 6B). TLE individuals exhibited alterations in sFNC and dFNC states, whereas no significant alteration has been observed in dFNC (STD) (Fig. 6). The sFNC significantly decreases in both GM and WM FNs specifically between GM1–GM4, GM4–GM5, GM10–GM11, GM11–WM1, GM11–WM4, WM2–GM1, WM4–WM9, and WM10–GM1. In contrast, it shows significant increases primarily within GM networks, including GM1–GM9, GM5–GM8, GM5–GM10, GM8–GM10, as well as between GM10–WM4 and GM8–WM4. However, the state analysis reveals that the FCs between TO - LOC (GM3) and ISFG - IFOC - PG(GM5), SPL - PoCG - PrCG (GM9) and CC - FM - SACR - rSLF - lTSG (WM4), and lILF (WM9) and H (WM10) increased in state 4 significantly (Fig. 9B) in individuals with TLE. The FCs between rAG - rMFG - rMTG (GM8) and lILF (WM9), and rAG - rMFG - rMTG (GM8) and lTSG - lSLF - CC(lS) (WM7) decreased significantly in state 4 in TLE (Fig. 9B). This implies that apart from the GM-FNs, WM-FNs are also associated with alterations in FC in the TLE group. In state analysis, the GM network, rAG - rMFG - rMTG played an important role with WM FNs. The state analysis also revealed that, in addition to GM–GM connections, GM–WM and WM–WM connections play a crucial role in the changes observed in TLE, underscoring the importance of both GM–WM and WM–WM interactions. The sFNC exhibits a negative correlation with dFNC in states 2, 3, 4 and 6 (Fig. 7). Among these, state 4, which shows the weakest negative correlation with sFNC, displays significantly altered connections in TLE. This suggests that states with low negative correlations with sFNC are particularly susceptible to alterations in TLE, even when such connections are not significant in the sFNC. This indicates that sFNC may not always be reliable for capturing group differences, as its variations could be influenced by differences in wakefulness levels across groups. This issue was addressed by investigating dFNC states, as group differences in connectivity are computed from similar dFNC states obtained through data-driven clustering (Damaraju et al. (2014)). The average connectivity of the state 4 does not change much but differ in terms of individual connections (Fig.S2), and alterations do not occur in states with higher positive or negative connectivity patterns. Although the connections show significant alterations in states 4, respectively (Fig. 9), they do not fall within the strongest 1% of connections (Fig. 8). This suggests that, while the average connections may not be the strongest, they can still differ significantly between TLE and HC.

From time frame contributions, it was found that significant alterations occurred in the states where a moderate number of FNC frames contributed (Fig. 10). The self-transition probability, i.e., state transition probability from one state to the same state, is higher for all the groups (HC, lTLE, and rTLE), but at state 4, the self-transition probability is less in HC than in lTLE and rTLE (Fig. 11). One of the reasons for the high self-transition is that the shifting interval is 1 TR in the sliding window method. However, the high self-transition does not imply no interstate transitions or a lack of dynamics in FC. While there are transitions between states, once a transition occurs, the FNC frame tends to stay in the new state for some time. Most rs-fMRI studies implicitly assume that all subjects are in similar states of wakefulness, regardless of the group, but this assumption has not been well validated (Liu and Falahpour (2020). The observation implies that state 4 is less dominant in HC, and in this state, the connectivity (GM3 - GM5, GM8 - WM9, GM8 - WM7, GM9 - WM4, WM9 - WM10) was altered significantly. Similarly, the state transition probability of HC differs from rTLE in the state 4 (see Fig. 11). The state-changing probability is higher for the HC group (Fig. S3), which implies that the rate of changing the network connectivity configuration is high in HC individuals.

The pattern of the states obtained in the dFNC analysis was different from the sFNC, which implies that the functional coordination between networks (GM-FNs or WM-FNs) varies over time and are designated as different subsystems. For instance, states 5 and 4 were dominated by negative connectivity, and state 1 was dominated by positive connectivity (Fig.S2). State 1 is more similar to the sFNC pattern (Fig. 7). Therefore, the sFNC can be viewed as the average behavior of discrete subsystems i.e. states all of which may not be very similar to the sFNC and the states could provide a detailed insight of the FNC configurations across time maintaining the stationary assumption.

The key finding of this study is that the dFNC analysis was able to capture FC changes more commonly identified among patients with TLE and not present among HC. These FC changes may be responsible for the network activities giving rise to chronic epilepsy. One potential network phenomenon that shares temporal and spatial characteristics with these dFNC changes is interictal epileptiform discharges (IED) (Alarcon et al. (1997); de Curtis and Avanzini (2001)). The presence of IED is an electrographic hallmark of the epileptic tissue in the brain. Among patients with epilepsy, including those with TLE, their properties are commonly used to monitor the disease activities, especially their response to both medical and surgical treatments (Alarcon et al. (1997); Chvojka et al. (2021)). The spatial distribution of IEDs helps to define the epileptic focus, which is vital for the surgical treatment (Drane et al. (2016)). Currently, invasive monitoring with the implantation of intracranial electrodes is required to capture the spatial distribution of the IEDs accurately. If the dFNC can be demonstrated to be a neuroimaging surrogate for IEDs, it may have the potential to replace invasive monitoring and spare these patients a surgical procedure.

## 5 Conclusion

In this study, we investigate whole-brain FC at both the GM and WM network levels. We examined whether the dynamic properties changed in patients with TLE. Our primary findings were that the FNC alteration was dynamic and state-dependent. This study was helpful in detecting significantly altered interactions between networks (GM and WM) in TLE.

## Supplementary Material

Supplementary Files

This is a list of supplementary files associated with this preprint. Click to download.

• SupplementaryMaterial.pdf

## Figures and Tables

**Table 1:** All GM and WM functional networks (FNs) and regions corresponding to the FNs in the brain.

Networks		Regions in the brain

GM	GM1	Precuneous cortex (PC), cingulate gyrus (CG), lateral occipital cortex (LOC)
	GM2	Occipital pole (OP)
	GM3	Temporooccipital (TO), lateral occipital cortex (LOC)
	GM4	Left angular gyrus (lAG), inferior temporal gyrus (ITG)
	GM5	Superior frontal gyrus (lSFG), left frontal orbital cortex (lFOC), paracingulate gyrus (PG)
	GM6	Left Heschl’s gyrus (lHG), left parietal operculum cortex (lPOC), left insular cortex (lIC), left superior temporal gyrus (lSTG), left post central gyrus (lPCG)
	GM7	Left middle frontal gyrus (lMFG), left angular gyrus (lAG), left inferior temporal gyrus (lITG)
	GM8	Right angular gyrus (rAG), right middle frontal gyrus (rMFG), right middle temporal gyrus (rMTG)
	GM9	Superior parietal lobule (SPL), postcentral gyrus (PoCG), precentral gyrus (PrCG)
	GM10	Right Heschl’s gyrus (rHG), right parietal operculum cortex (rPOC), right insular cortex (rIC), right superior temporal gyrus (rSTG), right supramarginal gyrus (rSMG), right precentral gyrus (rPrCG)
	GM11	Lingual gyrus (LG), temporal occipital fusiform cortex (TOFC)

WM	WM1	Putamen (P), caudate body (CB)
	WM2	Brain stem (BS)
	WM3	Right inferior longitudinal fasciculus (rILF)
	WM4	Body of the corpus callosum (CC(B)), right splenium of the corpus callosum (rCC(S)), genu of the corpus callosum (CC(G)), forceps minor (FM), superior and anterior corona radiata (SACR), right superior longitudinal fasciculus (rSLF), left temporal sub gyral (lTSG)
	WM5	Amygdala (A)
	WM6	Medial frontal gyrus (MFG)
	WM7	Left temporal sub gyral (lTSG), left superior longitudinal fasciculus (lSLF), left splenium of the corpus callosum (CC(lS))
	WM8	Midbrain (MB)
	WM9	Left inferior longitudinal fasciculus (lILF)
	WM10	Hippocampus (H)
	WM11	Thalamus (T), left pallidum (lPa)

The abbreviations of the regions are also given.

## Footnotes

1
https://osf.io/exbt4/

2
www.fil.ion.ucl.ac.uk/spm
